# Risks and benefits of oral modified‐release compared with oral immediate‐release opioid use after surgery: a systematic review and meta‐analysis

**DOI:** 10.1111/anae.16085

**Published:** 2023-07-06

**Authors:** S. Liu, A. Athar, D. Quach, A. E. Patanwala, J. M. Naylor, J. A. Stevens, N. Levy, R. D. Knaggs, D. N. Lobo, J. Penm

**Affiliations:** ^1^ Faculty of Medicine and Health School of Pharmacy, University of Sydney Sydney NSW Australia; ^2^ Department of Pharmacy Prince of Wales Hospital, Randwick Sydney NSW Australia; ^3^ Faculty of Medicine and Health, School of Medicine University of Notre Dame Sydney NSW Australia; ^4^ Faculty of Medical and Health Sciences, School of Pharmacy University of Auckland Auckland New Zealand; ^5^ Faculty of Medicine and Health, School of Pharmacy University of Sydney Sydney NSW Australia; ^6^ Department of Pharmacy Royal Prince Alfred Hospital Camperdown NSW Australia; ^7^ Whitlam Orthopaedic Research Centre, Orthopaedic Department, Liverpool Hospital Liverpool NSW Australia; ^8^ South Western Sydney Clinical School University of New South Wales Sydney NSW Australia; ^9^ School of Clinical Medicine, St VincentTable s Clinical Campus University of New South Wales Sydney NSW Australia; ^10^ University of Notre Dame Sydney NSW Australia; ^11^ Department of Anaesthesia and Perioperative Medicine West Suffolk Hospital Bury St. Edmunds UK; ^12^ School of Pharmacy University of Nottingham, and Primary Integrated Community Services Nottingham UK; ^13^ Nottingham Digestive Diseases Centre and National Institute for Health Research Nottingham Biomedical Research Centre Nottingham University Hospitals and University of Nottingham, Queen's Medical Centre Nottingham UK; ^14^ David Greenfield Metabolic Physiology Unit, MRC Versus Arthritis Centre for Musculoskeletal Ageing Research School of Life Sciences University of Nottingham, Queen's Medical Centre Nottingham UK; ^15^ Faculty of Medicine and Health, School of Pharmacy University of Sydney Sydney NSW Australia

**Keywords:** acute pain, modified‐release, opioid, surgery

## Abstract

Prescription of modified‐release opioids for acute postoperative pain is widespread despite evidence to show their use may be associated with an increased risk of adverse effects. This systematic review and meta‐analysis aimed to examine the available evidence on the safety and efficacy of modified‐release, compared with immediate‐release, oral opioids for postoperative pain in adults. We searched five electronic databases from 1 January 2003 to 1 January 2023. Published randomised clinical trials and observational studies on adults who underwent surgery which compared those who received oral modified‐release opioids postoperatively with those receiving oral immediate‐release opioids were included. Two reviewers independently extracted data on the primary outcomes of safety (incidence of adverse events) and efficacy (pain intensity, analgesic and opioid use, and physical function) and secondary outcomes (length of hospital stay, hospital readmission, psychological function, costs, and quality of life) up to 12 months postoperatively. Of the eight articles included, five were randomised clinical trials and three were observational studies. The overall quality of evidence was low. Modified‐release opioid use was associated with a higher incidence of adverse events (n = 645, odds ratio (95%CI) 2.76 (1.52–5.04)) and worse pain (n = 550, standardised mean difference (95%CI) 0.2 (0.04–0.37)) compared with immediate‐release opioid use following surgery. Our narrative synthesis concluded that modified‐release opioids showed no superiority over immediate‐release opioids for analgesic consumption, length of hospital stay, hospital readmissions or physical function after surgery. One study showed that modified‐release opioid use is associated with higher rates of persistent postoperative opioid use compared with immediate‐release opioid use. None of the included studies reported on psychological function, costs or quality of life.

## Introduction

Prescription of modified‐release opioid analgesics for acute postoperative pain is widespread. Since their introduction in the late 1990s [1], modified‐release opioid use has increased to account for over 30% of all opioids prescribed after surgery [2]. Modified‐release opioids were introduced into peri‐operative clinical practice where there was a dearth of effective analgesic techniques, reliance on unimodal analgesia (including as‐required intramuscular morphine, patient‐controlled analgesia and epidurals) and minimal use of simple analgesics (including non‐steroidal anti‐inflammatory drugs and paracetamol). The introduction of oral opioids coupled with the administration of non‐opioid analgesics allowed shorter lengths of stay and cost savings [3, 4]. The subsequent embedding of modified‐release opioids into routine clinical practice was driven by beliefs that: they provided greater and sustained pain relief; produced fewer ‘peak and trough’ serum opioid concentrations, leading to a lower risk of opioid dependence; and reduced nursing workload by reducing the frequency of analgesic dosing compared with immediate‐release opioid formulations [4, 5].

Emerging evidence now suggests that the use of modified‐release opioids for acute postoperative pain may be associated with more harm than benefit. Modified‐release opioid use for acute postoperative pain is associated with a greater risk of opioid‐related adverse events, in particular opioid‐induced ventilatory impairment [6, 7, 8, 9]. Due to their slow onset and offset, titration of the dose of modified‐release opioids is difficult, resulting in a sustained duration of any adverse effects encountered [10]. Existing studies indicate that modified‐release opioids yield less effective pain relief compared with immediate‐release opioids, and that a higher cumulative dose may be required [8, 11]. Modified‐release opioids have also been associated with prolonged hospital stay [2]. Since 2017, there has been irrefutable evidence that the use of modified‐release opioids is one of the main risk factors for persistent opioid use [12], with a recent study by Lam et al. providing further evidence of this [13]. Increasing awareness of the potential harms associated with modified‐release opioid use prompted the release of guidelines advising against the use of modified‐release opioids for the management of acute pain internationally [14, 15, 16, 17].

Within these guidelines, modified‐release opioids are not recommended for acute pain unless there is a demonstrable need or in exceptional circumstances [17]. However, there remains a paucity of literature to describe the circumstances in which modified or immediate‐release opioids may be most appropriate for the management of acute postoperative pain. Existing systematic reviews comparing modified‐release with immediate‐release opioids are largely limited to cancer‐related pain or chronic pain contexts [18, 19]. Furthermore, previous studies showing positive outcomes associated with modified‐release opioid use largely involve patient‐controlled analgesia as the comparator group, whereby improvements in physical function and reduced length of stay are attributable to removing the need for intravenous access and connection to a patient‐controlled analgesia pump [20]. There remains a research gap on the risks and benefits of modified‐release opioids compared with immediate‐release oral opioids for acute pain. Therefore, this systematic review and meta‐analysis aimed to examine the available evidence on their safety and efficacy for acute postoperative pain.

## Methods

This review was performed in adherence to the Preferred Reporting Items for Systematic Reviews and Meta‐Analyses (PRISMA) guidelines [21]. Original peer‐reviewed randomised clinical trials and observational studies were included. Studies involving adults (aged 18 years or older) who had undergone surgery were included. To ensure study findings were relevant to contemporary practice, we included articles published within the previous two decades. Articles written in languages other than English, conference abstracts, retracted articles and all other study types (including case reports, case series, editorials, expert opinion, literature reviews and qualitative studies) were excluded. Studies in which opioids were used exclusively for cancer‐related pain, palliative care, opioidsubstitution therapy (e.g. methadone oral liquid or sublingual buprenorphine), or for indications other than analgesia were excluded as they are outside the scope of this review. Modified‐release opioid formulations were defined as any opioid formulation with a mechanism designed to produce a sustained delivery or release rate of the active ingredient (such as modified‐release tablets) [22]. We included studies involving the use of oral modified‐release opioids for the management of postoperative pain. The comparator group(s) included oral immediate‐release opioid use for the management of postoperative pain. We did not include studies in which the comparator group(s) involved placebo or usual care only (where the proportion of patients taking immediate‐release opioid analgesia was <50% of the comparator group or was not specified).

The primary outcomes of this review included safety (incidence of adverse events including opioid‐induced ventilatory impairment [23] as defined by the study) and efficacy (pain intensity, quantity of analgesic use including opioid use in oral morphine milligram equivalents as an indicator of pain intensity, and physical function) as reported by individual studies, up to 12 months postoperatively. Secondary outcomes were length of hospital stay; hospital readmission rate; incidence of persistent postoperative opioid use (defined as opioid use for postoperative pain at 90 days or more after surgery [23]); psychological functioning; and costs and quality of life collected at up to 12 months after surgery.

We conducted a systematic search of five electronic databases for material published between 1 January 2003 and 1 January 2023. The full search strategy is available in online Supporting Information Table S1. After the removal of duplicates, two authors (AA and DQ) independently filtered articles by title and abstract for potentially eligible studies. Full‐text articles were then assessed independently by the same two authors to confirm eligibility. They also independently extracted data using a standard data extraction form including details of participants, study design, intervention method and duration and treatment outcomes. Discrepancies were discussed with other team members (SL and JP) to reach consensus. Where necessary, authors of included studies were contacted to obtain further data not present in the published articles.

Quality assessment of all included studies was conducted by two authors (SL and AA) independently. Randomised controlled trials were assessed using the Revised Cochrane Risk of Bias Assessment Tool for randomised trials [24] and non‐randomised studies were assessed using the Risk of Bias in Non‐randomised Studies of Interventions [25]. Two authors (SL and JN) used the GRADE component to categorise the quality and strength of the evidence reported in randomised controlled trials included in the meta‐analysis as very low, low, moderate or high for the primary outcomes (incidence of opioid‐related adverse events and pain intensity) [26]. Discrepancies were resolved by consensus between two authors (SL and JN). A GRADE assessment checklist was used to ensure consistency and reproducibility (online Supporting Information Table S2) [27] and findings were summarised using the GRADEpro software (https://gradepro.org).

Data were synthesised from all included studies (randomised controlled trials and non‐randomised studies) for safety outcomes and from included randomised controlled trials only for efficacy outcomes.

Safety and efficacy outcomes reported in included randomised controlled trials were considered separately for meta‐analysis. We used odds ratios with 95%CI for categorical safety outcomes and standardised mean differences (Hedges' g) with 95%CI for continuous efficacy outcomes with different units. We calculated the I^2^ statistic to assess statistical heterogeneity among included studies. Heterogeneity was evaluated according to definitions in the Cochrane Handbook [28]. One study was not included in the meta‐analysis as information on safety and efficacy outcomes required to pool data was not available [29]. One study was removed from the meta‐analysis of efficacy outcomes [30] and one was removed from the safety outcomes [31] due to significant methodological heterogeneity in the pain intensity and adverse event outcomes reported, respectively. The mean (SD) values reported in the study by Park et al. were reversed between modified‐release and immediate‐release groups to account for the direction of effect [32]. A random effects model with inverse variance was used for all meta‐analyses [33]. We planned to explore publication bias using funnel plots if there were 10 or more studies included in the meta‐analysis, but the number of studies included did not reach this. The a priori α level used was 0.05 for all analyses. RevMan software (version 5.4, Copenhagen, Denmark) was used to compile data and perform statistical analyses.

## Results

**Figure 1:**
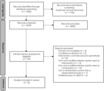
Study flow diagram.

The search strategy yielded 3660 articles, of which 59 full‐text articles were assessed for eligibility. Refinement using the inclusion and exclusion criteria resulted in eight studies eligible for inclusion [13, 29, 30, 31, 32, 34, 35, 36] (Fig. 1). There were five randomised controlled trials [29, 30, 31, 32, 36] and three observational studies [13, 34, 35] included. Of the five randomised controlled trials, four [30, 31, 32, 36] were included in the meta‐analysis.

Two studies were performed in Germany [35, 36], two in Australia [13, 31] and the remainder in a range of countries including the USA [34], South Korea [32] and Canada [29]. Four studies involved oxycodone [30, 31, 34, 35], one involved morphine [36], and three involved other opioids including codeine [29], tramadol [32] and all opioids (Table 1) [13].

**Table 1:** Summary of included studies reporting on the safety and effect of modified‐release opioid use after surgery.

Author, Funding	Study design, Study size: modified‐release opioid group vs. comparison group, Surgery performed	Analgesics: modified‐release opioid group vs. comparison group	Outcomes (modified‐release opioid group vs. comparison group)				
			Safety (adverse events)	Effectiveness/efficacy			Other (incidence of PPOU, length of hospital stay, hospital readmission, psychological function, costs, quality of life)
				Pain intensity	Analgesic use	Physical function	
Chung et al. [29] Study partially supported by grant from Purdue Pharma (Canada) Inc.	Double‐blind, randomised controlled trial n = 42 vs. 42 Laparoscopic cholecystectomy	MR oral codeine 150 mg twice daily on POD 1, then 100 mg twice daily on POD 2 regularly vs. IR oral codeine 60 mg with paracetamol 600 mg 6‐hourly on POD 1, then codeine 30 mg with paracetamol 300 mg 6‐hourly on POD 2 regularly	No significant difference in incidence of adverse events (incidence NR, p = NR).	No significant difference in reduction in mean VAS pain intensity from baseline between groups (30 minutes after the first dose 29.5% vs. 29.4%, p = 0.7, other time‐points p = NR).	No significant differences for daily dose (p = 0.4) or number of doses of rescue fentanyl per day (p = 0.5).	No significant difference in physical function between groups (p = 0.7).	Length of stay: no significant difference between groups (p = NR). Hospital readmission: no significant difference between groups (p = NR).
Kogan et al. [30] No funding declared.	Double‐blind randomised controlled trial n = 60 vs. 60 Elective coronary artery bypass graft	MR oral oxycodone 10 mg twice daily regularly vs. IR oral oxycodone 5 mg four times daily regularly	Overall: 20% vs. 11.7% (p = NR) Somnolence: 11.7% vs. 2.2% (p = 0.01) No significant difference for nausea (23.3% vs. 26.7%, p = 0.52), pruritus (3.3% vs. 3.3%, p = NR), vomiting (3.3% vs. 5%, p = 0.31), dizziness (1.7% vs. 1.7%, p = NR), headache (1.7% vs. 0%, p = NR) and atrial fibrillation (20% vs. 18.3%, p = 0.26).	MR mean VAS pain scores were significantly higher (p < 0.05) at all time points, including at 24 h postoperatively: 2.2 (0.4) vs. 1.1 (0.4) (p < 0.05).	Total mean morphine equivalent use: 9.5 (5.6) vs. 8.8 (5.1) mg (p = 0.09) MR had more rescue oxycodone IR (p = 0.01) and paracetamol (p = 0.05) at all time‐points	NR	Length of hospital stay: 4.1 (1.3) vs. 4.5 (1.8) days (p = 0.3)
Park et al. [32] Two authors are employees of Janssen Korea.	Double‐blind randomised controlled trial n = 161 vs. 153 Total knee arthroplasty	MR oral tramadol 150 mg with 1300 mg paracetamol 12‐hourly regularly vs. IR oral tramadol 75 mg with 650 mg paracetamol 6‐hourly regularly	Discontinuation due to adverse event: 9.9% vs. 3.3% (p = 0.018). Nausea: 49.7% vs. 44.4% (p = NR). Vomiting: 28% vs. 24.2% (p = NR). Constipation: 24.2% vs. 19.6% (p = NR).	MR opioid group reported less pain relief than the IR opioid group: SPID at 24‐h post‐administration 14.7 (15.3) vs. 18.1 (12.5) (p = 0.034). Mean difference in SPID at 24 h post‐administration −3.37 (one‐sided 97.5% CI −6.47 to −0.26).	No significant difference in the mean frequency or dosage of rescue medication used between groups (p > 0.05).	NR	NR
Schoenwald et al. [31] Funding from the Queensland University of Technology Scholarship	Double‐blind randomised controlled trial n = 66 vs. 65 Elective caesarean section	MR oral oxycodone 10 mg twice daily regularly vs. IR oral oxycodone 20 mg daily dose (divided into 3 doses) when required	No significant difference for nausea (U = 1333, p = 0.8), itching (U = 1367, p = 0.9) or drowsiness (U = 1326, p = 0.7).	No significant difference for VAS pain at rest (95% CI −4.8 to 11.9 mm, p = 0.40) or during sitting/movement (95%CI ‐15.2–8.3 mm, p = 0.6) over 24 h. Median (IQR) total Brief Pain Inventory pain interference (higher score indicating more pain interference): 25 (11–39) vs. 15 (8–34) (p = 0.03).	Median (IQR) oxycodone dose over 28 h: 30 (30–40) mg vs. 25 (20–35) mg (p = 0.001)^a^ No difference for rescue tramadol use (U = 1565, p = 1.0)	NR	NR
Scholz et al. [36] Grünenthal funded and designed the trial and analysed and interpreted the data. Several authors are employees of Grünenthal.	Double‐blind randomised controlled trial n = 50 vs. 161 Primary unilateral bunionectomy	MR oral morphine 60 mg single dose vs. IR oral cebranopadol 200 μg, 400 μg or 600 μg single dose	Total treatment‐emergent adverse events: 92% vs. 76% (p = NR).	Mean (SD) sum of pain intensity using NRS for first 2–10 h for MR morphine 43.8 (22.5) vs. ‐ cebranopadol 600 μg: vs. 37.0 (22.9) (p = 0.04). ‐ cebranopadol 400 μg: 36.6 (19.3) (p = 0.06). ‐ cebranopadol 200 μg: 47.8 (19.6) (p = 0.2).	Median time to first paracetamol use: MR morphine 4.2 h vs. 7.2 h (p = NR). Mean (SD) amount of paracetamol used in first 24 h: MR morphine 1320.0 (978.1) mg vs. ‐ cebranopadol 600 μg: 1219.3 (881.4) mg ‐ cebranopadol 400 μg: 1281.3 (972.5) mg ‐ cebranopadol 200 μg: 1490.6 (890.6) mg (p = NR).	NR	NR
Kerpsack et al. [34] No funding declared.	Prospective observational study n = 57 vs. 59 Primary total knee or hip arthroplasty	MR oral oxycodone 20 mg to 40 mg (frequency NR) vs. IR oral oxycodone 10 mg with paracetamol (dose NR) 4‐hourly regularly	NR	Total McGill Short Form Pain Questionnaire total score on morning of POD 1: 9.8 vs. 12.0 (p = 0.03). VAS pain intensity on morning of POD 2: 4.0 vs. 3.5 (p = 0.02). No significant differences between groups at all other time‐points (p = NR).	No significant difference for mean number of breakthrough doses between groups (2.6 vs. 3.2, p = 0.6).	NR	NR
Lam et al. [13] Funded by untied educational grant from Seqirus Pty Ltd.	Retrospective observational study n = 43,473 vs. 79,363 (opioid naïve 31,952 vs. 66,485 opioid experienced 11,521 vs. 12,878) All surgical procedures	Any oral MR opioid ± oral IR opioid prescribed after hospital discharge vs. Any IR oral opioid prescribed after hospital discharge	NR	NR	NR	NR	Incidence of PPOU: ‐ At 3 months after surgery among opioid naïve patients: 4.8% vs. 1.3% (p = NR) ‐ At 6 months after surgery among opioid naïve patients 0.9% vs. 0.3% (p = NR) ‐ At 3 months after surgery among opioid experienced patients 32.6% vs. 23.7% (p = NR) ‐ At 6 months after surgery among opioid experienced patients 19.7% vs. 15.4% (p = NR)
Oppermann et al. [35] Study designed and conducted under sponsorship by Mundipharma Research GmbH & Co KG, Limburg an der Lahn, Germany	Prospective observational study n = 43 vs. 37 Primary total knee arthroplasty	MR oral oxycodone/naloxone 10/5 mg twice daily regularly vs. IR piritramide, IR tramadol, or IR oxycodone (dose, route, frequency NR)	Overall: 23% vs. 38% (p = NR). No significant difference for bowel function index (p = NR).	No significant difference for Brief Pain Inventory Short‐Form scores between groups (p > 0.001).	Mean (SD) daily oxycodone dose: 23.3 (15.3) mg vs. 19.9 (8.3) mg (p = NR). Rescue opioid analgesic use: 39.5% vs. 86.5% (p = NR).	MR oxycodone/naloxone group had higher modified Larson function score (p = 0.02). No significant differences for Hospital for Special Surgery score (p = 0.19) or ability to attend physical therapy (p = NR).	NR

PPOU, persistent postoperative opioid use; MR, modified‐release; POD, postoperative day; IR, immediate‐release; NR, not reported; VAS, visual analogue scale; SPID, sum of pain intensity difference; NRS, numerical rating scale.

^a^ Values confirmed with corresponding author of study.

Of the five randomised controlled trials, two were graded as having some concerns of bias [29, 31] and three as having a low risk of bias [30, 32, 36]. Two trials contained some concerns of bias arising from the selection of reported results because pre‐specified analysis plans were not reported [29, 31]. Of the three observational studies, all were graded as having a moderate risk of bias due to potential confounding and bias in the measurement of outcomes as outcome assessors were not blinded (online Supporting Information Table S3) [13, 34, 35].

**Figure 2:**
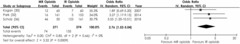
Incidence of opioid‐related adverse events during inpatient stay among participants given modified‐release opioids compared with immediate‐release opioids in sensitivity analysis articles with low risk of bias. MR, modified‐release; IR, immediate‐release; IV, Random, inverse variance random effects model used.

Of the eight included studies, six reported the incidence of opioid‐related adverse events after surgery [29, 30, 31, 32, 35, 36], including five randomised controlled trials [29, 30, 31, 32, 36] and one observational study [35]. Three randomised controlled trials were included in the meta‐analysis on safety outcomes (Table 2, Fig. 2) [30, 32, 36]. For the remaining three studies [29, 31, 35], a brief qualitative summary is provided.

**Table 2:** GRADE assessment for safety and efficacy outcomes associated with modified‐release compared with immediate‐release opioid use following surgery.

Outcomes	No. of participants	Certainty of the evidence, GRADE component^a^	Effect estimates	
			Relative (95%CI)	Absolute (95%CI)
Incidence of opioid‐related adverse events during inpatient stay	645 (3 RCTs)	⨁⨁◯◯ Low^b^ ^,^ ^c^	OR 2.76 (1.52–5.04)	248 more per 1000 (101 more–379 more)
Pain intensity 0–24 h postoperatively	550 (3 RCTs)	⨁⨁◯◯ Low^b^ ^,^ ^c^ ^,^ ^d^ ^,^ ^e^	N/A	SMD 0.2 higher (0.04 higher–0.37 higher)

RCT, randomised controlled trial; N/A, not applicable; SMD, standardised mean difference.

^a^ High, high confidence that the true effect lies close to that of the estimate of the effect; moderate, moderate confidence that the true effect is likely to be close to the estimate of the effect, but with a possibility that it is substantially different; low, confidence and the true effect may be substantially different from the estimate of the effect; very low, very little confidence and the true effect is likely to be substantially different from estimate of effect.

^b^ Some studies only measured this outcome during hospital inpatient stay. Given modified‐release opioids are often supplied following hospital discharge after surgery, this outcome timeframe may be insufficient to detect outcomes occurring after hospital discharge.

^c^ Two authors in Park et al. [33] were employees of Janssen Korea. The study by Scholz et al. [38] was funded, designed and analysed by Grünenthal.

^d^ Pain intensity may be a subjective outcome measure (measured using visual analogue scale or numeric rating scale).

^e^ Pain outcomes related to the surgical populations in the included studies may not be applicable to other contexts.

Meta‐analysis of three randomised controlled trials showed participants (n = 645) who received modified‐release opioids following surgery had 2.76‐fold higher odds (95% CI 1.52–5.04, p = 0.0009) of experiencing an opioid‐related adverse event during inpatient stay compared with those receiving immediate‐release opioids only (Fig. 2). Based on GRADE assessment, there is limited confidence that this estimate of effect lies close to the true effect due to an insufficient timeframe of outcome measurement and potential publication bias related to pharmaceutical‐industry funding (Table 2). Minimal heterogeneity was found across studies reporting safety outcomes (Fig. 2).

Of the three studies not included in the meta‐analysis, two were randomised controlled trials [29, 31] and one was an observational study [35]. All three studies reported no difference in the incidence of opioid‐related adverse events between patients given modified‐release or immediate‐release opioids after surgery (Table 1) [29, 31, 35].

Data were synthesised from included randomised controlled trials only for the efficacy outcomes. All five randomised controlled trials included reported efficacy outcomes after surgery [29, 30, 31, 32, 36]. Pain intensity was reported in five trials [29, 30, 31, 32, 36], quantity of opioid use in five trials [29, 30, 31, 32, 36], and physical function in one trial [29].

**Figure 3:**
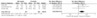
Pain intensity over the first 24 h postoperatively among participants given modified‐release opioids compared with immediate‐release opioids in sensitivity analysis articles with low risk of bias. MR, modified‐release; IR, immediate‐release; IV, Random, inverse variance random effects model used.

Three randomised controlled trials were included in the meta‐analysis on pain intensity following surgery (Table 2, Fig 3) [31, 32, 36]. A brief qualitative summary is provided for the remaining two trials [29, 30].

Meta‐analysis of three randomised controlled trials showed participants (n = 550) who received modified‐release opioids following surgery experienced worse pain during the first 24 h postoperatively compared with those given immediate‐release opioids only, with an absolute standardised mean difference (95%CI) in pain scores of 0.2 (0.04–0.37, p = 0.02) higher in the modified‐release opioid group over this period (Fig. 3). Based on the GRADE component, there is low confidence that this estimate of effect lies close to the true effect due to subjectivity in the chosen outcomes and an insufficient timeframe for outcome measurement, potential publication bias related to pharmaceutical industry funding and limited applicability of pain outcomes to surgical contexts outside of the procedures reported in the included trials (Table 2). Minimal heterogeneity was found across studies reporting safety outcomes (Fig. 3).

Of the two randomised controlled trials not included in the meta‐analysis [29, 30], one reported that modified‐release oxycodone use was associated with higher visual analogue scale pain scores at all time‐points compared with those patients given immediate‐release oxycodone after elective coronary artery bypass graft (p < 0.05) [30]. The other randomised controlled trial reported no difference in pain intensity between modified‐release and immediate‐release opioid groups following laparoscopic cholecystectomy (Table 1) [29].

Of five trials reporting on the quantity of opioid use after surgery [29, 30, 31, 32, 36], three examined opioid dose [29, 30, 31] and five examined rescue analgesia use [29, 30, 31, 32, 36]. Due to significant heterogeneity in reporting of opioid or rescue analgesic use between included trials, a meta‐analysis was not conducted.

One randomised controlled trial reported higher oxycodone use among patients given modified‐release compared with immediate‐release oxycodone after elective caesarean section, with a median (IQR) oxycodone dose over 28 h of 30 (30–40) mg vs. 25 (20–35) mg (p = 0.001) [31]. The remaining two trials reported no significant difference in the number of opioids used between modified‐release and immediate‐release opioid groups [29, 30].

Two trials reported a higher quantity of rescue analgesia used among the modified‐release compared with the immediate‐release opioid group [30, 36]. One trial reported higher quantities of immediate‐release oxycodone (p = 0.01) and paracetamol (p = 0.05) used at all time points after elective coronary artery bypass graft [30] among participants given modified‐release compared with immediate‐release oxycodone after surgery. Another trial reported participants given modified‐release morphine used higher quantities of paracetamol, with a mean (SD) paracetamol use over 24 h after primary unilateral bunionectomy of 1320.0 (978.1) mg compared with 1219.3 (881.4) mg among those given immediate‐release opioids (statistical significance not reported) [36].

Three trials reported no difference in the quantity of rescue analgesics used between modified‐release and immediate‐release opioid groups (Table 1) [29, 31, 32].

One trial allocated 84 participants randomly to receive modified‐release or immediate‐release codeine after laparoscopic cholecystectomy and reported no difference in physical function between groups (p = 0.7) [29].

Two double‐blind randomised controlled trials reported on the length of hospital stay and hospital readmission rates [29, 30]. Both reported no difference in length of hospital stay [30] or hospital readmission rates [29, 30] between modified‐release and immediate‐release opioid groups (Table 1). One retrospective cohort study examined the incidence of persistent postoperative opioid use among 92,863 surgical patients following hospital discharge [13]. Patients given modified‐release opioids following surgery had a higher incidence of persistent opioid use at 3 months (opioid naïve 4.8% vs. 1.3%; opioid experienced 32.6% vs. 23.7%, statistical significance not reported) and 6 months (opioid naïve 0.9% vs. 0.3%; opioid experienced 19.7% vs. 15.4%, statistical significance not reported) postoperatively compared with those given only immediate‐release opioids after surgery (Table 1) [13]. No studies reported on psychological function, costs or quality of life outcomes.

## Discussion

This systematic review and meta‐analysis identified eight studies examining the safety and efficacy of oral modified‐release opioids compared with oral immediate‐release opioids after surgery. Based on low‐quality evidence, modified‐release opioid use was associated with a higher incidence of opioid‐related adverse events and worse pain compared with immediate‐release opioid use only following surgery. Modified‐release opioids showed no superiority over immediate‐release opioids for analgesic consumption or physical function after surgery. Few studies reported on length of hospital stay and readmission rates after discharge and, where included, no significant differences between groups overall were observed. One large observational study showed that modified‐release opioid use is associated with a higher incidence of persistent postoperative opioid use compared with immediate‐release opioid use only after surgery [13]. No studies examined differences in the incidence of opioid‐induced ventilatory impairment, psychological functioning, costs or quality of life associated with the use of modified vs. immediate‐release opioids after surgery.

There is increasing evidence to suggest that the harms associated with modified‐release opioid use for acute postoperative pain may outweigh the potential benefits. The manufacturers of modified‐release opioid formulations such as modified‐release oxycodone in Australia [37], the UK [38] and the USA [39] explicitly advise against use within the first 24 h after surgery, first 12–24 h after surgery and immediate postoperative periods, respectively. Previous systematic reviews assessing the safety and efficacy of modified‐release opioid preparations have largely focused on cancer‐related pain and chronic non‐cancer pain [18, 19]. A systematic review performed by Mesgarpour et al. in 2013 examining nine studies involving modified‐release opioids for the management of cancer‐related pain showed no significant differences in safety or efficacy among patients given modified‐release compared with immediate‐release opioids [18]. Another systematic review of six studies by Pedersen et al. published in 2014 reported that modified‐release opioid use in the context of chronic non‐cancer pain was associated with no difference in pain relief, rescue analgesia consumption, the incidence of adverse events, sleep quality or physical function compared with immediate‐release opioid use [19]. Therefore, the existing literature on the use of modified‐release opioids in other contexts aligns with our findings that modified‐release opioids do not provide superior pain relief, physical function or analgesic use outcomes compared with immediate‐release opioids. Our systematic review adds to the literature by highlighting that modified‐release opioid use in the context of acute postoperative pain is associated with a higher incidence of opioid‐related adverse events during the hospital stay and persistent postoperative opioid use, and worse pain compared with immediate‐release opioids. This aligns with, and provides support for, existing international guidelines discouraging the prescription of modified‐release opioids for acute postoperative pain [14, 15, 16, 17, 40]. Given modified‐release opioids provide limited benefits over immediate‐release opioids and are associated with an increased risk of harm when used after surgery, they should not be used for the management of acute postoperative pain in adults. In light of these findings, revision of guidelines that continue to recommend the use of modified‐release opioids for postoperative analgesia [41, 42] may be required.

Of the included studies, many failed to report a pre‐specified analysis plan or adequately blind patients and investigators, which led to an increased risk of bias. The overall quality of the evidence was low. Further high‐quality studies examining the safety and efficacy of modified‐release compared with immediate‐release opioid use after surgery are warranted to validate our findings. Future studies should consider comprehensive reporting of outcomes in line with existing literature to enhance generalisability and facilitate comparison against other studies. Limited studies reported on the length of hospital stay, readmission rates and persistent postoperative opioid use, and no studies examined the incidence of opioid‐induced ventilatory impairment, costs, psychological functioning or quality of life associated with the use of oral modified‐release compared with immediate‐release opioids after surgery. This represents an avenue for future research to strengthen the available evidence in this area.

A study describing the use of modified‐release opioids for postoperative pain has since been retracted as the data had been falsified [43] and was not eligible for inclusion in this review. Furthermore, five [13, 29, 32, 35, 36] of the eight studies included in the present review declared affiliations or funding from the pharmaceutical industry. A previous systematic review demonstrated that pharmaceutical industry sponsorship is associated with an increased likelihood of outcomes that are favourable to the funder than studies with other sponsors [44]. The impact of research funding by the pharmaceutical industry on study outcomes related to modified‐release opioid use remains unknown. Further investigation is warranted to examine the impact of the retracted or pharmaceutical industry‐sponsored articles describing modified‐release opioid use on the literature and subsequent effects on health policy and clinical practice.

There are several limitations to this study. Although we performed a rigorous search across five electronic databases, the search strategy was limited to articles written in the English language. Thus, relevant articles in other languages may have been excluded. Grey literature was not searched, and this may introduce a degree of publication bias. There was variability in opioid dosing frequency between studies, where opioid dosing on a regular or as‐required basis may produce different serum opioid concentrations postoperatively, leading to variability in outcomes between studies. Given that pain scores are non‐parametric outcomes, reporting of the median (IQR) is more appropriate than reporting of the mean (SD). However, several of the included studies reported pain scores using mean and SD, which may compromise the reliability of the findings of individual studies and thus the review as a whole. The definition of opioid‐related adverse events varied between studies, which limits the accuracy and reliability of the aggregated incidence of adverse events across studies. Furthermore, the incidence of persistent postoperative opioid use was only reported in one study, which limits the reliability of conclusions regarding this outcome. The study findings may not be generalisable to surgery types that were not included in the review. Thus, the safety and efficacy of modified‐release opioids in patients undergoing these operations remain unknown. Finally, several studies showed some concerns of bias and the overall quality of the evidence was low. This may reduce confidence that the reported findings reflect the true effect of the interventions for these studies.

## Supporting information


**Table S1.** Full search strategy.
**Table S2.** GRADE assessment checklist of studies included in the meta‐analysis.
**Table S3.** Risk of bias assessments of the included studies.
