# Supplementary material for: Risks and benefits of oral modified‐release compared with oral immediate‐release opioid use after surgery: a systematic review and meta‐analysis
Source: Anaesthesia. 2023 Jul 6;78(10):1225–36. doi: 10.1111/anae.16085 (PMC10952256; doi:10.1111/anae.16085)
Supplement: Supplementary file 1 — Table S1. Full search strategy. Table S2. GRADE assessment checklist of studies included in the meta‐analysis. Table S3. Risk of bias assessments of the included studies. [file ANAE-78-1225-s001.docx]

**Table S1** Full search strategy

| Medline (1960 to Present, OvidSP)   1. Opioid*.mp. or exp Analgesics, Opioid/ 2. Narcotic*.mp or exp Narcotics/ 3. Opiate*.mp. or exp Opiate Alkaloids/ 4. (alfentanil or alphaprodine or acetyldihydrocodeine or allylprodine or alphamethylfentanyl or buprenorphine or butorphanol or benzylmorphine or betaprodine or bremazocine or codeine or contin or dextromoramide or dextropropoxyphene or dezocine or diacetylmorphine or diamorphine or dihydrocodeine or dihydromorphone or dipipanone or dihydromorphine or diphenoxylate or enadoline or ethylketazocine or ethylmorphine or etorphine or etonitazene or fentanyl or heroin or hydromorphine or hydrocodone or hydromorphone or ketazocine or ketobemidone or lefetamine or levorphanol or levomethadon or levomethadyl or levomethorphan* or loperamide or meperidine or meptazinol or methadone or methadyl or morphine or nalbuphine or narcotic* or nicocodeine or nicomorphine or normorphine or noscapine or opiate* or opium or oxycodone or oxymorphone or ohmefentanyl or opium or oripavine or oxycontinpapaveretum or pentazocine or paperverin or phenazocine or percocet or peronine or pethidine or phencyclidine or pholcodine or piritramid or phenoperidine or pirinitramide or promedol or prodine or propoxyphene or remifentanil or sufentanil or tapentadol or tilidine or thebaine or tramadol).mp. 5. 1 or 2 or 3 or 4 6. exp Delayed-Action Preparations/ 7. (slow-releas* or controlled-releas* or delayed-release or delayed-action* or depot* or extended-releas* or prolonged-releas* or prolonged-action* or sustained-releas* or timed-releas* or patch* or slow releas* or controlled releas* or delayed release or delayed action* or depot* or extended releas* or prolonged releas* or prolonged action* or sustained releas* or timed releas* or patch*).mp. 8. 6 or 7 9. Ambulatory Surgical Procedures/ 10. exp Surgical Procedures, Operative/ 11. exp Specialties, Surgical/ 12. Pain, Postoperative/ 13. (ambulatory or postoperat* or emergenc* or sudden onset or surgical* or surger* or fracture* or hospitali?ation* or postop* or trauma* or injur* or breakthrough* or dental or labo?r* or childbirth* or c?esarean).mp. 14. 9 or 10 or 11 or 12 or 13 15. 5 and 8 and 14 16. Limit 15 to (English language and humans) |
| --- |

**Table S2** GRADE assessment checklist of studies included in the meta-analysis.

| **Checklist for the Quality Assessment Tool for Safety outcomes (incidence of adverse events)– studies by Kogan et al., Park et al., and Scholz et al.**  **Study limitations (Risk of Bias) – Kogan et al.**  1) Was random sequence generation used (i.e. no potential for selection bias)?   - Yes   2) Was allocation concealment used (i.e. no potential for selection bias)?   - Yes   3) Was there blinding of participants and personnel (i.e. no potential for performance bias)?   - Yes   4) Was there blinding of outcome assessment (i.e. no potential for detection bias)?   - No   5) Was an objective outcome used?   - Yes   6) Were more than 80%^^[[1]](#footnote-1)^^ of participants enrolled in trials included in the analysis (i.e. no potential reporting bias)?   - Yes   7) Were data reported consistently for the outcome of interest (i.e., no potential selective reporting)?   - Yes   8) No other biases reported? (i.e. no potential of other bias)   - Yes   9) Did the trials end as scheduled (i.e not stopped early)?   - Yes   **Study limitations (Risk of Bias) – Park et al.**  1) Was random sequence generation used (i.e. no potential for selection bias)?   - Yes   2) Was allocation concealment used (i.e. no potential for selection bias)?   - Yes   3) Was there blinding of participants and personnel (i.e. no potential for performance bias)?   - Yes   4) Was there blinding of outcome assessment (i.e. no potential for detection bias)?   - Yes   5) Was an objective outcome used?   - Yes   6) Were more than 80%^^[[2]](#footnote-2)^^ of participants enrolled in trials included in the analysis (i.e. no potential reporting bias)?   - Yes   7) Were data reported consistently for the outcome of interest (i.e., no potential selective reporting)?   - Yes   8) No other biases reported? (i.e. no potential of other bias)   - Yes   9) Did the trials end as scheduled (i.e not stopped early)?   - Yes   No  **Study limitations (Risk of Bias) – Scholz et al.**  1) Was random sequence generation used (i.e. no potential for selection bias)?   - Yes   2) Was allocation concealment used (i.e. no potential for selection bias)?   - Yes   3) Was there blinding of participants and personnel (i.e. no potential for performance bias)?   - Yes   4) Was there blinding of outcome assessment (i.e. no potential for detection bias)?   - Yes   5) Was an objective outcome used?   - Yes   6) Were more than 80%^^[[3]](#footnote-3)^^ of participants enrolled in trials included in the analysis (i.e. no potential reporting bias)?   - Yes   7) Were data reported consistently for the outcome of interest (i.e., no potential selective reporting)?   - Yes   8) No other biases reported? (i.e. no potential of other bias)   - Yes   9) Did the trials end as scheduled (i.e not stopped early)?   - Yes   **Inconsistency^^[[4]](#footnote-4)^^**  1) Point estimates did not vary widely?   - Yes   2) To what extent did confidence intervals overlap?   - Some overlap   (confidence intervals overlap but not all overlap at least one point estimate)  3) Was the direction of effect consistent?   - Yes   4) What was the magnitude of statistical heterogeneity (as measured by I^2^)?   - Low (e.g. I^2^ <40%)   5) Was the test for heterogeneity statistically significant (p<0.1)?   - Not statistically significant   **Indirectness**  1) Were the populations in included studies applicable to the decision context?   - Applicable (the rate of opioid-related adverse events is not expected to differ depending on the type of surgery performed)   2) Were the interventions in the included studies applicable to the decision context?   - Applicable   3) Was the included outcome not a surrogate outcome?   - Yes   4) Was the outcome timeframe sufficient?   - Insufficient – some studies only collected adverse events occurring during hospital stay. Some adverse events may not be detected until after hospital discharge (e.g., constipation). Given usual care involves the provision of modified-release opioids following hospital discharge, a longer time frame for outcome measurement is required.   5) Were the conclusions based on direct comparisons?   - Yes   **Imprecision**  1) Was the confidence interval for the pooled estimate not consistent with benefit and harm?   - Yes   2) What is the magnitude of the median sample size?   - Intermediate (e.g. 100-300 participants)   3) What was the magnitude of the number of included studies?   - Small (e.g. <5 studies)   4) Was the outcome a common event (e.g. occurs more than 1/100)?   - Yes   *Further optional question for those engaged in guideline development^^[[5]](#footnote-5)^^*  5) Was there no evidence of serious harm associated with treatment?   - Not applicable   **Publication Bias (other considerations)**  1) Did the authors conduct a comprehensive search?   - Yes   2) Did the authors search for grey literature?   - No   3) Authors did not apply restrictions to study selection on the basis of language?   - No (English language only)   4) There was no industry influence on studies included in the review?   - Two authors of the article by Park and colleagues were employees of Janssen Korea. The study by Scholz and colleagues was funded, designed, and analysed by Gruenthal.   5) There was no evidence of funnel plot asymmetry?   - Unclear (Funnel plot not examined as there were less than ten studies included in the meta-analysis)   6) There was no discrepancy in findings between published and unpublished trials?   - Unclear |
| --- |
| **Checklist for the Quality Assessment Tool for Efficacy outcomes (pain intensity)– studies by Park et al., Schoenwald et al., and Scholz et al.**  **Study limitations (Risk of Bias) – Park et al.**  1) Was random sequence generation used (i.e. no potential for selection bias)?   - Yes   2) Was allocation concealment used (i.e. no potential for selection bias)?   - Yes   3) Was there blinding of participants and personnel (i.e. no potential for performance bias)?   - Yes   4) Was there blinding of outcome assessment (i.e. no potential for detection bias)?   - Yes   5) Was an objective outcome used?   - No (pain intensity measured on an 11 point numeric rating scale)   6) Were more than 80%^^[[6]](#footnote-6)^^ of participants enrolled in trials included in the analysis (i.e. no potential reporting bias)?   - Yes   7) Were data reported consistently for the outcome of interest (i.e., no potential selective reporting)?   - Yes   8) No other biases reported? (i.e. no potential of other bias)   - Yes   9) Did the trials end as scheduled (i.e not stopped early)?   - Yes   **Study limitations (Risk of Bias) – Schoenwald et al.**  1) Was random sequence generation used (i.e. no potential for selection bias)?   - Yes   2) Was allocation concealment used (i.e. no potential for selection bias)?   - Yes   3) Was there blinding of participants and personnel (i.e. no potential for performance bias)?   - Yes   4) Was there blinding of outcome assessment (i.e. no potential for detection bias)?   - Yes   5) Was an objective outcome used?   - No (pain intensity measured on a visual analogue scale)   6) Were more than 80%^^[[7]](#footnote-7)^^ of participants enrolled in trials included in the analysis (i.e. no potential reporting bias)?   - Yes   7) Were data reported consistently for the outcome of interest (i.e., no potential selective reporting)?   - Yes   8) No other biases reported? (i.e. no potential of other bias)   - Yes   9) Did the trials end as scheduled (i.e not stopped early)?   - Yes   **Study limitations (Risk of Bias) – Scholz et al.**  1) Was random sequence generation used (i.e. no potential for selection bias)?   - Yes   2) Was allocation concealment used (i.e. no potential for selection bias)?   - Yes   3) Was there blinding of participants and personnel (i.e. no potential for performance bias)?   - Yes   4) Was there blinding of outcome assessment (i.e. no potential for detection bias)?   - Yes   5) Was an objective outcome used?   - No (pain intensity measured on an 11 point numeric rating scale)   6) Were more than 80%^^[[8]](#footnote-8)^^ of participants enrolled in trials included in the analysis (i.e. no potential reporting bias)?   - Yes   7) Were data reported consistently for the outcome of interest (i.e., no potential selective reporting)?   - Yes   8) No other biases reported? (i.e. no potential of other bias)   - Yes   9) Did the trials end as scheduled (i.e not stopped early)?   - Yes   **Inconsistency^^[[9]](#footnote-9)^^**  1) Point estimates did not vary widely?   - No   2) To what extent did confidence intervals overlap?   - Some overlap   (confidence intervals overlap but not all overlap at least one point estimate)  3) Was the direction of effect consistent?   - Yes   4) What was the magnitude of statistical heterogeneity (as measured by I^2^)?   - Low (e.g. I^2^ <40%)   5) Was the test for heterogeneity statistically significant (p<0.1)?   - Not statistically significant   **Indirectness**  1) Were the populations in included studies applicable to the decision context?   - Not applicable – surgical procedures ranged from bunionectomy, caesarean section, to total knee arthroplasty. Pain trajectories and opioid requirements may differ between these procedures and the decision context.   2) Were the interventions in the included studies applicable to the decision context?   - Applicable   3) Was the included outcome not a surrogate outcome?   - Yes   4) Was the outcome timeframe sufficient?   - Insufficient – studies only collected pain scores during hospital stay. Given usual care involves the provision of modified-release opioids following hospital discharge, a longer time frame for outcome measurement is required.   5) Were the conclusions based on direct comparisons?   - Yes   **Imprecision**  1) Was the confidence interval for the pooled estimate not consistent with benefit and harm?   - No   2) What is the magnitude of the median sample size?   - Intermediate (e.g. 100-300 participants)   3) What was the magnitude of the number of included studies?   - Small (e.g. <5 studies)   4) Was the outcome a common event (e.g. occurs more than 1/100)?   - Yes   *Further optional question for those engaged in guideline development^^[[10]](#footnote-10)^^*  5) Was there no evidence of serious harm associated with treatment?   - Not applicable   **Publication Bias (other considerations)**  1) Did the authors conduct a comprehensive search?   - Yes   2) Did the authors search for grey literature?   - No   3) Authors did not apply restrictions to study selection on the basis of language?   - No (English only)   4) There was no industry influence on studies included in the review?   - Two authors of the article by Park and colleagues were employees of Janssen Korea. The study by Scholz and colleagues was funded, designed, and analysed by Gruenthal.   5) There was no evidence of funnel plot asymmetry?   - Unclear (Funnel plot not examined as there were less than ten studies included in the meta-analysis)   6) There was no discrepancy in findings between published and unpublished trials?   - Unclear |

**Table S3** Risk of bias assessments of the included studies.

Controlled-release codeine is equivalent to acetaminophen plus codeine for post-cholecystectomy analgesia, Chung et al., 2004

| **Chung et al., 2004** | | | |
| --- | --- | --- | --- |
| **Domain 1: Risk of bias arising from the randomization process** | **Signalling questions** | **Comments** | **Response options** |
|  | **1.1 Was the allocation sequence random?** | Study blinding was maintained using the double-dummy technique, with matching placebo. Patients were randomized to receive either active controlled-release codeine | Y |
|  | **1.2 Was the allocation sequence concealed until participants were enrolled and assigned to interventions?** |  | Y |
|  | **1.3 Did baseline differences between intervention groups suggest a problem with the randomization process?** | Similar in demographic characteristics. | PN |
|  | **Risk-of-bias judgement** | Per algorithm | Low risk |

| **Chung et al., 2004** | | | |
| --- | --- | --- | --- |
| **Domain 2: Risk of bias due to deviations from the intended interventions (effect of assignment to intervention)** | **Signalling questions** | **Comments** | **Response options** |
|  | **2.1. Were participants aware of their assigned intervention during the trial?** | Study blinding was maintained using the double-dummy technique, with matching placebo | N |
|  | **2.2. Were carers and people delivering the interventions aware of participants' assigned intervention during the trial?** |  | PN |
|  | **2.3. If Y/PY/NI to 2.1 or 2.2: Were there deviations from the intended intervention that arose because of the trial context?** |  | NA |
|  | **2.4 If Y/PY to 2.3: Were these deviations likely to have affected the outcome?** |  | NA |
|  | **2.5. If Y/PY/NI to 2.4: Were these deviations from intended intervention balanced between groups?** |  | NA |
|  | **2.6 Was an appropriate analysis used to estimate the effect of assignment to intervention?** |  | PY |
|  | **2.7 If N/PN/NI to 2.6: Was there potential for a substantial impact (on the result) of the failure to analyse participants in the group to which they were randomized?** |  | NA |
|  | **Risk-of-bias judgement** | Per algorithm | Low risk |

| **Chung et al., 2004** | | | |
| --- | --- | --- | --- |
| **Domain 2: Risk of bias due to deviations from the intended interventions (*effect of adhering to intervention*)** | **Signalling questions** | **Comments** | **Response options** |
|  | **2.1. Were participants aware of their assigned intervention during the trial?** |  | N |
|  | **2.2. Were carers and people delivering the interventions aware of participants' assigned intervention during the trial?** |  | PN |
|  | **2.3. [If applicable:] If Y/PY/NI to 2.1 or 2.2: Were important non-protocol interventions balanced across intervention groups?** |  | NA |
|  | **2.4. [If applicable:] Were there failures in implementing the intervention that could have affected the outcome?** |  | NA |
|  | **2.5. [If applicable:] Was there non-adherence to the assigned intervention regimen that could have affected participants’ outcomes?** |  | NA |
|  | **2.6. If N/PN/NI to 2.3, or Y/PY/NI to 2.4 or 2.5: Was an appropriate analysis used to estimate the effect of adhering to the intervention?** |  | NA |
|  | **Risk-of-bias judgement** | Per algorithm | Low risk |

| **Chung et al., 2004** | | | |
| --- | --- | --- | --- |
| **Domain 3: Missing outcome data** | **Signalling questions** | **Comments** | **Response options** |
|  | **3.1 Were data for this outcome available for all, or nearly all, participants randomized?** |  | PN |
|  | **3.2 If N/PN/NI to 3.1: Is there evidence that the result was not biased by missing outcome data?** |  | N |
|  | **3.3 If N/PN to 3.2: Could missingness in the outcome depend on its true value?** |  | NI |
|  | **3.4 If Y/PY/NI to 3.3: Is it likely that missingness in the outcome depended on its true value?** |  | PN |
|  | **Risk-of-bias judgement** | Per algorithm | Some concerns |

| **Chung et al., 2004** | | | |
| --- | --- | --- | --- |
| **Domain 4: Risk of bias in measurement of the outcome** | **Signalling questions** | **Comments** | **Response options** |
|  | **4.1 Was the method of measuring the outcome inappropriate?** | Appropriate scales used. | N |
|  | **4.2 Could measurement or ascertainment of the outcome have differed between intervention groups?** | Specified time points used. | N |
|  | **4.3 If N/PN/NI to 4.1 and 4.2: Were outcome assessors aware of the intervention received by study participants?** |  | N |
|  | **4.4 If Y/PY/NI to 4.3: Could assessment of the outcome have been influenced by knowledge of intervention received?** |  | NA |
|  | **4.5 If Y/PY/NI to 4.4: Is it likely that assessment of the outcome was influenced by knowledge of intervention received?** |  | NA |
|  | **Risk-of-bias judgement** | Per algorithm | Low risk |

| **Chung et al., 2004** | | | |
| --- | --- | --- | --- |
|  | **Signalling questions** | **Comments** | **Response options** |
| **Domain 5: Risk of bias in selection of the reported result** | **5.1 Were the data that produced this result analysed in accordance with a pre-specified analysis plan that was finalized before unblinded outcome data were available for analysis?** | Not reported | NI |
|  | **Is the numerical result being assessed likely to have been selected, on the basis of the results, from...** |  |  |
|  | **5.2. ... multiple eligible outcome measurements (e.g. scales, definitions, time points) within the outcome domain?** |  | N |
|  | **5.3 ... multiple eligible analyses of the data?** |  | N |
|  | **Risk-of-bias judgement** |  | Some concerns |

| **Overall risk of bias**  **Chung et al., 2004** | **Risk-of-bias judgement** |  | Some concerns |
| --- | --- | --- | --- |

Early oral analgesia after fast-track cardiac anesthesia, Kogan et al., 2007

| **Kogan et al., 2007** | | | |
| --- | --- | --- | --- |
| **Domain 1: Risk of bias arising from the randomization process** | **Signalling questions** | **Comments** | **Response options** |
|  | **1.1 Was the allocation sequence random?** | Yes (instructions were placed in envelopes).  Allocation sequence was concealed as instructions were placed in in sealed envelopes, mixed, and randomly assigned. | Y |
|  | **1.2 Was the allocation sequence concealed until participants were enrolled and assigned to interventions?** |  | Y |
|  | **1.3 Did baseline differences between intervention groups suggest a problem with the randomization process?** | No | N |
|  | **Risk-of-bias judgement** | Per algorithm | Low risk |

| **Kogan et al., 2007** | | | |
| --- | --- | --- | --- |
| **Domain 2: Risk of bias due to deviations from the intended interventions (effect of assignment to intervention)** | **Signalling questions** | **Comments** | **Response options** |
|  | **2.1. Were participants aware of their assigned intervention during the trial?** | All staff, except one of the investigators (N.P) were blinded to the results of randomisation. | N |
|  | **2.2. Were carers and people delivering the interventions aware of participants' assigned intervention during the trial?** |  | PY |
|  | **2.3. If Y/PY/NI to 2.1 or 2.2: Were there deviations from the intended intervention that arose because of the trial context?** |  | N |
|  | **2.4 If Y/PY to 2.3: Were these deviations likely to have affected the outcome?** |  | NA |
|  | **2.5. If Y/PY/NI to 2.4: Were these deviations from intended intervention balanced between groups?** |  | NA |
|  | **2.6 Was an appropriate analysis used to estimate the effect of assignment to intervention?** |  | Y |
|  | **2.7 If N/PN/NI to 2.6: Was there potential for a substantial impact (on the result) of the failure to analyse participants in the group to which they were randomized?** |  | N |
|  | **Risk-of-bias judgement** | Per algorithm | Low risk |

| **Kogan et al., 2007** | | | |
| --- | --- | --- | --- |
| **Domain 2: Risk of bias due to deviations from the intended interventions (*effect of adhering to intervention*)** | **Signalling questions** | **Comments** | **Response options** |
|  | **2.1. Were participants aware of their assigned intervention during the trial?** | All staff, except one of the investigators (N.P) were blinded to the results of randomisation. | N |
|  | **2.2. Were carers and people delivering the interventions aware of participants' assigned intervention during the trial?** |  | PY |
|  | **2.3. [If applicable:] If Y/PY/NI to 2.1 or 2.2: Were important non-protocol interventions balanced across intervention groups?** |  | Y |
|  | **2.4. [If applicable:] Were there failures in implementing the intervention that could have affected the outcome?** |  | NA |
|  | **2.5. [If applicable:] Was there non-adherence to the assigned intervention regimen that could have affected participants’ outcomes?** |  | NA |
|  | **2.6. If N/PN/NI to 2.3, or Y/PY/NI to 2.4 or 2.5: Was an appropriate analysis used to estimate the effect of adhering to the intervention?** |  | NA |
|  | **Risk-of-bias judgement** | Per algorithm | Low risk |

| **Kogan et al., 2007** | | | |
| --- | --- | --- | --- |
| **Domain 3: Missing outcome data** | **Signalling questions** | **Comments** | **Response options** |
|  | **3.1 Were data for this outcome available for all, or nearly all, participants randomized?** |  | Y |
|  | **3.2 If N/PN/NI to 3.1: Is there evidence that the result was not biased by missing outcome data?** |  | NA |
|  | **3.3 If N/PN to 3.2: Could missingness in the outcome depend on its true value?** |  | NA |
|  | **3.4 If Y/PY/NI to 3.3: Is it likely that missingness in the outcome depended on its true value?** |  | NA |
|  | **Risk-of-bias judgement** | Per algorithm | Low risk |

| **Kogan et al., 2007** | | | |
| --- | --- | --- | --- |
| **Domain 4: Risk of bias in measurement of the outcome** | **Signalling questions** | **Comments** | **Response options** |
|  | **4.1 Was the method of measuring the outcome inappropriate?** | The primary outcome was measured using the visual analogue scale which appear appropriate. | N |
|  | **4.2 Could measurement or ascertainment of the outcome have differed between intervention groups?** |  | N |
|  | **4.3 If N/PN/NI to 4.1 and 4.2: Were outcome assessors aware of the intervention received by study participants?** | All staff, except one of the investigators (N.P) were blinded to the results of randomisation. | PY |
|  | **4.4 If Y/PY/NI to 4.3: Could assessment of the outcome have been influenced by knowledge of intervention received?** | Not reported, but one of the investigators was aware of results of randomisation and therefore not blinded. | Nil |
|  | **4.5 If Y/PY/NI to 4.4:** **Is it likely that assessment of the outcome was influenced by knowledge of intervention received?** |  | PN |
|  | **Risk-of-bias judgement** | Per algorithm | Some concerns |

| **Kogan et al., 2007** | | | |
| --- | --- | --- | --- |
|  | **Signalling questions** | **Comments** | **Response options** |
| **Domain 5: Risk of bias in selection of the reported result** | **5.1 Were the data that produced this result analysed in accordance with a pre-specified analysis plan that was finalized before unblinded outcome data were available for analysis?** | Pre-specified protocol used | Y |
|  | **Is the numerical result being assessed likely to have been selected, on the basis of the results, from...** |  |  |
|  | **5.2. ... multiple eligible outcome measurements (e.g. scales, definitions, time points) within the outcome domain?** |  | N |
|  | **5.3 ... multiple eligible analyses of the data?** |  | N |
|  | **Risk-of-bias judgement** |  | Low risk |

| **Overall risk of bias**  **Kogan et al., 2007** | **Risk-of-bias judgement** |  | Low risk |
| --- | --- | --- | --- |

A randomized study to compare the efficacy and safety of extended-release and immediate-release tramadol HCl/acetaminophen in patients with acute pain following total knee replacement, Park et al., 2014

| **Park et al., 2014** | | | |
| --- | --- | --- | --- |
| **Domain 1: Risk of bias arising from the randomization process** | **Signalling questions** | **Comments** | **Response options** |
|  | **1.1 Was the allocation sequence random?** | During the double-blind treatment period (48 hours from study group assignment) patients were randomized in a 1:1 ratio to the study drug treatment group (TA-ER group) or the comparator treatment group (TA-IR group) and administered the assigned agent over a period of 48 hours. This period included patients who completed the 48 hour assessment as well as those who discontinued at an earlier time point. A double dummy method was used to preserve blinding. | Y |
|  | **1.2 Was the allocation sequence concealed until participants were enrolled and assigned to interventions?** |  | PY |
|  | **1.3 Did baseline differences between intervention groups suggest a problem with the randomization process?** |  | N |
|  | **Risk-of-bias judgement** | Per algorithm | Low risk |

| **Park et al., 2014** | | | |
| --- | --- | --- | --- |
| **Domain 2: Risk of bias due to deviations from the intended interventions (effect of assignment to intervention)** | **Signalling questions** | **Comments** | **Response options** |
|  | **2.1. Were participants aware of their assigned intervention during the trial?** | Double blinded. | N |
|  | **2.2. Were carers and people delivering the interventions aware of participants' assigned intervention during the trial?** |  | N |
|  | **2.3. If Y/PY/NI to 2.1 or 2.2: Were there deviations from the intended intervention that arose because of the trial context?** |  | NA |
|  | **2.4 If Y/PY to 2.3: Were these deviations likely to have affected the outcome?** |  | NA |
|  | **2.5. If Y/PY/NI to 2.4: Were these deviations from intended intervention balanced between groups?** |  | NA |
|  | **2.6 Was an appropriate analysis used to estimate the effect of assignment to intervention?** |  | Y |
|  | **2.7 If N/PN/NI to 2.6: Was there potential for a substantial impact (on the result) of the failure to analyse participants in the group to which they were randomized?** |  | NA |
|  | **Risk-of-bias judgement** | Per algorithm | Low risk |

| **Park et al., 2014** | | | |
| --- | --- | --- | --- |
| **Domain 2: Risk of bias due to deviations from the intended interventions (*effect of adhering to intervention*)** | **Signalling questions** | **Comments** | **Response options** |
|  | **2.1. Were participants aware of their assigned intervention during the trial?** | Double blinded. | N |
|  | **2.2. Were carers and people delivering the interventions aware of participants' assigned intervention during the trial?** |  | N |
|  | **2.3. [If applicable:] If Y/PY/NI to 2.1 or 2.2: Were important non-protocol interventions balanced across intervention groups?** |  | NA |
|  | **2.4. [If applicable:] Were there failures in implementing the intervention that could have affected the outcome?** |  | NA |
|  | **2.5. [If applicable:] Was there non-adherence to the assigned intervention regimen that could have affected participants’ outcomes?** |  | NA |
|  | **2.6. If N/PN/NI to 2.3, or Y/PY/NI to 2.4 or 2.5: Was an appropriate analysis used to estimate the effect of adhering to the intervention?** |  | NA |
|  | **Risk-of-bias judgement** | Per algorithm | Low risk |

| **Park et al., 2014** | | | |
| --- | --- | --- | --- |
| **Domain 3: Missing outcome data** | **Signalling questions** | **Comments** | **Response options** |
|  | **3.1 Were data for this outcome available for all, or nearly all, participants randomized?** |  | N |
|  | **3.2 If N/PN/NI to 3.1: Is there evidence that the result was not biased by missing outcome data?** | LOCF method applied. | Y |
|  | **3.3 If N/PN to 3.2: Could missingness in the outcome depend on its true value?** |  | NA |
|  | **3.4 If Y/PY/NI to 3.3: Is it likely that missingness in the outcome depended on its true value?** |  | NA |
|  | **Risk-of-bias judgement** | Per algorithm | Low risk |

| **Park et al., 2014** | | | |
| --- | --- | --- | --- |
| **Domain 4: Risk of bias in measurement of the outcome** | **Signalling questions** | **Comments** | **Response options** |
|  | **4.1 Was the method of measuring the outcome inappropriate?** | Appropriate scales used. | N |
|  | **4.2 Could measurement or ascertainment of the outcome have differed between intervention groups?** |  | N |
|  | **4.3 If N/PN/NI to 4.1 and 4.2: Were outcome assessors aware of the intervention received by study participants?** |  | N |
|  | **4.4 If Y/PY/NI to 4.3: Could assessment of the outcome have been influenced by knowledge of intervention received?** |  | NA |
|  | **4.5 If Y/PY/NI to 4.4: Is it likely that assessment of the outcome was influenced by knowledge of intervention received?** |  | NA |
|  | **Risk-of-bias judgement** | Per algorithm | Low risk |

| **Park et al., 2014** | | | |
| --- | --- | --- | --- |
|  | **Signalling questions** | **Comments** | **Response options** |
| **Domain 5: Risk of bias in selection of the reported result** | **5.1 Were the data that produced this result analysed in accordance with a pre-specified analysis plan that was finalized before unblinded outcome data were available for analysis?** | Registered with pre-set plan. | Y |
|  | **Is the numerical result being assessed likely to have been selected, on the basis of the results, from...** |  |  |
|  | **5.2. ... multiple eligible outcome measurements (e.g. scales, definitions, time points) within the outcome domain?** |  | N |
|  | **5.3 ... multiple eligible analyses of the data?** |  | N |
|  | **Risk-of-bias judgement** |  | Low risk |

| **Overall risk of bias**  **Park et al., 2014** | **Risk-of-bias judgement** |  | Low risk |
| --- | --- | --- | --- |

Nurse practitioner led pain management the day after caesarean section: A randomised controlled trial and follow-up study. Schoenwald et al., 2018

| **Schoenwald et al., 2018** | | | |
| --- | --- | --- | --- |
| **Domain 1: Risk of bias arising from the randomization process** | **Signalling questions** | **Comments** | **Response options** |
|  | **1.1 Was the allocation sequence random?** | The sequence was generated by an independent researcher and concealment of group allocation was based on the sequentially numbered opaque sealed envelopes technique. Each sequentially numbered envelope contained the treatment group allocation and instructions that remained concealed until given to the anaesthetist on the day of surgery. Envelopes were prepared by another independent researcher and the sequential number corresponded to the research participant number on the master allocation list (allocation ratio 1:1). | Y |
|  | **1.2 Was the allocation sequence concealed until participants were enrolled and assigned to interventions?** |  | Y |
|  | **1.3 Did baseline differences between intervention groups suggest a problem with the randomization process?** | Only statistical difference was ethnicity. More Caucasian participants in intervention group. | PN |
|  | **Risk-of-bias judgement** | Per algorithm | Low Risk |

| **Schoenwald et al., 2018** | | | |
| --- | --- | --- | --- |
| **Domain 2: Risk of bias due to deviations from the intended interventions (effect of assignment to intervention)** | **Signalling questions** | **Comments** | **Response options** |
|  | **2.1. Were participants aware of their assigned intervention during the trial?** | No, blinded. | N |
|  | **2.2. Were carers and people delivering the interventions aware of participants' assigned intervention during the trial?** |  | N |
|  | **2.3. If Y/PY/NI to 2.1 or 2.2: Were there deviations from the intended intervention that arose because of the trial context?** |  | NA |
|  | **2.4 If Y/PY to 2.3: Were these deviations likely to have affected the outcome?** |  | NA |
|  | **2.5. If Y/PY/NI to 2.4: Were these deviations from intended intervention balanced between groups?** |  | NA |
|  | **2.6 Was an appropriate analysis used to estimate the effect of assignment to intervention?** |  | Y |
|  | **2.7 If N/PN/NI to 2.6: Was there potential for a substantial impact (on the result) of the failure to analyse participants in the group to which they were randomized?** |  | NA |
|  | **Risk-of-bias judgement** | Per algorithm | Low Risk |

| **Schoenwald et al., 2018** | | | |
| --- | --- | --- | --- |
| **Domain 2: Risk of bias due to deviations from the intended interventions (*effect of adhering to intervention*)** | **Signalling questions** | **Comments** | **Response options** |
|  | **2.1. Were participants aware of their assigned intervention during the trial?** | Blinded RCT | N |
|  | **2.2. Were carers and people delivering the interventions aware of participants' assigned intervention during the trial?** |  | N |
|  | **2.3. [If applicable:] If Y/PY/NI to 2.1 or 2.2: Were important non-protocol interventions balanced across intervention groups?** |  | NA |
|  | **2.4. [If applicable:] Were there failures in implementing the intervention that could have affected the outcome?** |  | NA |
|  | **2.5. [If applicable:] Was there non-adherence to the assigned intervention regimen that could have affected participants’ outcomes?** |  | NA |
|  | **2.6. If N/PN/NI to 2.3, or Y/PY/NI to 2.4 or 2.5: Was an appropriate analysis used to estimate the effect of adhering to the intervention?** |  | NA |
|  | **Risk-of-bias judgement** | Per algorithm | Low risk |

| **Schoenwald et al., 2018** | | | |
| --- | --- | --- | --- |
| **Domain 3: Missing outcome data** | **Signalling questions** | **Comments** | **Response options** |
|  | **3.1 Were data for this outcome available for all, or nearly all, participants randomized?** |  | Y |
|  | **3.2 If N/PN/NI to 3.1: Is there evidence that the result was not biased by missing outcome data?** |  | NA |
|  | **3.3 If N/PN to 3.2: Could missingness in the outcome depend on its true value?** |  | NA |
|  | **3.4 If Y/PY/NI to 3.3: Is it likely that missingness in the outcome depended on its true value?** |  | NA |
|  | **Risk-of-bias judgement** | Per algorithm | Low risk |

| **Schoenwald et al., 2018** | | | |
| --- | --- | --- | --- |
| **Domain 4: Risk of bias in measurement of the outcome** | **Signalling questions** | **Comments** | **Response options** |
|  | **4.1 Was the method of measuring the outcome inappropriate?** | All methods of grading were supported, primary outcome was measured using the VAS pain score. | N |
|  | **4.2 Could measurement or ascertainment of the outcome have differed between intervention groups?** |  | N |
|  | **4.3 If N/PN/NI to 4.1 and 4.2: Were outcome assessors aware of the intervention received by study participants?** |  | N |
|  | **4.4 If Y/PY/NI to 4.3: Could assessment of the outcome have been influenced by knowledge of intervention received?** |  | NA |
|  | **4.5 If Y/PY/NI to 4.4: Is it likely that assessment of the outcome was influenced by knowledge of intervention received?** |  | NA |
|  | **Risk-of-bias judgement** | Per algorithm | Low risk |

| **Schoenwald et al., 2018** | | | |
| --- | --- | --- | --- |
|  | **Signalling questions** | **Comments** | **Response options** |
| **Domain 5: Risk of bias in selection of the reported result** | **5.1 Were the data that produced this result analysed in accordance with a pre-specified analysis plan that was finalized before unblinded outcome data were available for analysis?** | Not reported | NI |
|  | **Is the numerical result being assessed likely to have been selected, on the basis of the results, from...** |  |  |
|  | **5.2. ... multiple eligible outcome measurements (e.g. scales, definitions, time points) within the outcome domain?** |  | N |
|  | **5.3 ... multiple eligible analyses of the data?** |  | N |
|  | **Risk-of-bias judgement** |  | Some concerns |

| **Overall risk of bias**  **Schoenwald et al., 2018** | **Risk-of-bias judgement** |  | Some concerns |
| --- | --- | --- | --- |

Cebranopadol: A novel, first-in-class, strong analgesic: Results from a randomized phase iia clinical trial in postoperative acute pain

Scholz et al., 2018

| **Scholz et al., 2018** | | | |
| --- | --- | --- | --- |
| **Domain 1: Risk of bias arising from the randomization process** | **Signalling questions** | **Comments** | **Response options** |
|  | **1.1 Was the allocation sequence random?** | Randomisation was based on computer-generated randomization lists, provided by an external contract re- search organization. Investigators were given a unique series of numbers for assignment to each patient in ascending numerical order. Block randomization was applied, randomizing patients in a 1:1:1:1:1 ratio. Blinding of patients and investigators was achieved using a double-blind, double-dummy technique. | Y |
|  | **1.2 Was the allocation sequence concealed until participants were enrolled and assigned to interventions?** |  | Y |
|  | **1.3 Did baseline differences between intervention groups suggest a problem with the randomization process?** | Only statistical difference was ethnicity. | PN |
|  | **Risk-of-bias judgement** | Per algorithm | Low Risk |

| **Scholz et al., 2018** | | | |
| --- | --- | --- | --- |
| **Domain 2: Risk of bias due to deviations from the intended interventions (effect of assignment to intervention)** | **Signalling questions** | **Comments** | **Response options** |
|  | **2.1. Were participants aware of their assigned intervention during the trial?** | Blinding of patients and investigators was achieved using a double-blind, double-dummy technique.. | N |
|  | **2.2. Were carers and people delivering the interventions aware of participants' assigned intervention during the trial?** |  | N |
|  | **2.3. If Y/PY/NI to 2.1 or 2.2: Were there deviations from the intended intervention that arose because of the trial context?** |  | NA |
|  | **2.4 If Y/PY to 2.3: Were these deviations likely to have affected the outcome?** |  | NA |
|  | **2.5. If Y/PY/NI to 2.4: Were these deviations from intended intervention balanced between groups?** |  | NA |
|  | **2.6 Was an appropriate analysis used to estimate the effect of assignment to intervention?** |  | Y |
|  | **2.7 If N/PN/NI to 2.6: Was there potential for a substantial impact (on the result) of the failure to analyse participants in the group to which they were randomized?** |  | NA |
|  | **Risk-of-bias judgement** | Per algorithm | Low Risk |

| **Scholz et al., 2018** | | | |
| --- | --- | --- | --- |
| **Domain 2: Risk of bias due to deviations from the intended interventions (*effect of adhering to intervention*)** | **Signalling questions** | **Comments** | **Response options** |
|  | **2.1. Were participants aware of their assigned intervention during the trial?** | Blinding of patients and investigators was achieved using a double-blind, double-dummy technique. | N |
|  | **2.2. Were carers and people delivering the interventions aware of participants' assigned intervention during the trial?** |  | N |
|  | **2.3. [If applicable:] If Y/PY/NI to 2.1 or 2.2: Were important non-protocol interventions balanced across intervention groups?** |  | NA |
|  | **2.4. [If applicable:] Were there failures in implementing the intervention that could have affected the outcome?** |  | NA |
|  | **2.5. [If applicable:] Was there non-adherence to the assigned intervention regimen that could have affected participants’ outcomes?** |  | NA |
|  | **2.6. If N/PN/NI to 2.3, or Y/PY/NI to 2.4 or 2.5: Was an appropriate analysis used to estimate the effect of adhering to the intervention?** |  | NA |
|  | **Risk-of-bias judgement** | Per algorithm | Low risk |

| **Scholz et al., 2018** | | | |
| --- | --- | --- | --- |
| **Domain 3: Missing outcome data** | **Signalling questions** | **Comments** | **Response options** |
|  | **3.1 Were data for this outcome available for all, or nearly all, participants randomized?** |  | Y |
|  | **3.2 If N/PN/NI to 3.1: Is there evidence that the result was not biased by missing outcome data?** |  | NA |
|  | **3.3 If N/PN to 3.2: Could missingness in the outcome depend on its true value?** |  | NA |
|  | **3.4 If Y/PY/NI to 3.3: Is it likely that missingness in the outcome depended on its true value?** |  | NA |
|  | **Risk-of-bias judgement** | Per algorithm | Low risk |

| **Scholz et al., 2018** | | | |
| --- | --- | --- | --- |
| **Domain 4: Risk of bias in measurement of the outcome** | **Signalling questions** | **Comments** | **Response options** |
|  | **4.1 Was the method of measuring the outcome inappropriate?** | All methods of grading were supported, primary outcome was measured using the SPI scale. | N |
|  | **4.2 Could measurement or ascertainment of the outcome have differed between intervention groups?** |  | N |
|  | **4.3 If N/PN/NI to 4.1 and 4.2: Were outcome assessors aware of the intervention received by study participants?** |  | N |
|  | **4.4 If Y/PY/NI to 4.3: Could assessment of the outcome have been influenced by knowledge of intervention received?** |  | NA |
|  | **4.5 If Y/PY/NI to 4.4: Is it likely that assessment of the outcome was influenced by knowledge of intervention received?** |  | NA |
|  | **Risk-of-bias judgement** | Per algorithm | Low risk |

| **Scholz et al., 2018** | | | |
| --- | --- | --- | --- |
|  | **Signalling questions** | **Comments** | **Response options** |
| **Domain 5: Risk of bias in selection of the reported result** | **5.1 Were the data that produced this result analysed in accordance with a pre-specified analysis plan that was finalized before unblinded outcome data were available for analysis?** |  | Y |
|  | **Is the numerical result being assessed likely to have been selected, on the basis of the results, from...** |  |  |
|  | **5.2. ... multiple eligible outcome measurements (e.g. scales, definitions, time points) within the outcome domain?** |  | N |
|  | **5.3 ... multiple eligible analyses of the data?** |  | N |
|  | **Risk-of-bias judgement** |  | Low risk |

| **Overall risk of bias**  **Scholz et al., 2018** | **Risk-of-bias judgement** |  | Low risk |
| --- | --- | --- | --- |

The use of controlled-release versus scheduled oxycodone in the immediate postoperative period following total joint arthroplasty, Kerpsack et al., 2005

|  | **Signalling questions** | **Description** | **Response options** |
| --- | --- | --- | --- |
| **Bias due to confounding** | | | |
|  | 1.1 Is there potential for confounding of the effect of intervention in this study?  **If N/PN to 1.1:** the study can be considered to be at low risk of bias due to confounding and no further signalling questions need be considered |  | Y |
|  | **If Y/PY to 1.1**: determine whether there is a need to assess time-varying confounding: |  |  |
|  | 1.2. Was the analysis based on splitting participants’ follow up time according to intervention received?  **If N/PN**, answer questions relating to baseline confounding (1.4 to 1.6)  **If Y/PY**, go to question 1.3. |  | N |
|  | 1.3. Were intervention discontinuations or switches likely to be related to factors that are prognostic for the outcome?  **If N/PN**, answer questions relating to baseline confounding (1.4 to 1.6)  **If Y/PY**, answer questions relating to both baseline and time-varying confounding (1.7 and 1.8) |  | NA |

|  | **Questions relating to baseline confounding only** | | |
| --- | --- | --- | --- |
|  | 1.4. Did the authors use an appropriate analysis method that controlled for all the important confounding domains? | Restriction based on revision surgery, epidural anaesthesia, postoperative ketorolac use, use of PC pump, known allergy. | PN |
|  | 1.5. **If Y/PY to 1.4**: Were confounding domains that were controlled for measured validly and reliably by the variables available in this study? |  | NI |
|  | 1.6. Did the authors control for any post-intervention variables that could have been affected by the intervention? |  | N |
|  | **Questions relating to baseline and time-varying confounding** | |  |
|  | 1.7. Did the authors use an appropriate analysis method that controlled for all the important confounding domains and for time-varying confounding? |  | NI |
|  | 1.8. **If Y/PY to 1.7**: Were confounding domains that were controlled for measured validly and reliably by the variables available in this study? |  | NA |
|  | **Risk of bias judgement** |  | Moderate |

| **Bias in selection of participants into the study** | | | |
| --- | --- | --- | --- |
|  | 2.1. Was selection of participants into the study (or into the analysis) based on participant characteristics observed after the start of intervention?  **If N/PN to 2.1:** go to 2.4 | Selection not altered after start of intervention | N |
|  | 2.2. **If Y/PY to 2.1**: Were the post-intervention variables that influenced selection likely to be associated with intervention?  2.3 **If Y/PY to 2.2**: Were the post-intervention variables that influenced selection likely to be influenced by the outcome or a cause of the outcome? |  | NA  NA |
|  | 2.4. Do start of follow-up and start of intervention coincide for most participants? |  | Y |
|  | 2.5. **If Y/PY to 2.2 and 2.3, or N/PN to 2.4**: Were adjustment techniques used that are likely to correct for the presence of selection biases? |  | NA |
|  | **Risk of bias judgement** |  | Low |

| **Bias in classification of interventions** | | | |
| --- | --- | --- | --- |
|  | 3.1 Were intervention groups clearly defined? |  | Y |
|  | 3.2 Was the information used to define intervention groups recorded at the start of the intervention? |  | Y |
|  | 3.3 Could classification of intervention status have been affected by knowledge of the outcome or risk of the outcome? |  | PN |
|  | **Risk of bias judgement** |  | Low |

| **Bias due to deviations from intended interventions** | | | |
| --- | --- | --- | --- |
|  | **If your aim for this study is to assess the effect of assignment to intervention, answer questions 4.1 and 4.2** | |  |
|  | 4.1. Were there deviations from the intended intervention beyond what would be expected in usual practice? |  | N |
|  | 4.2. **If Y/PY to 4.1**: Were these deviations from intended intervention unbalanced between groups *and* likely to have affected the outcome? |  | NA |
|  | **If your aim for this study is to assess the effect of starting and adhering to intervention, answer questions 4.3 to 4.6** | |  |
|  | 4.3. Were important co-interventions balanced across intervention groups? |  | Y |
|  | 4.4. Was the intervention implemented successfully for most participants? |  | Y |
|  | 4.5. Did study participants adhere to the assigned intervention regimen? |  | Y |
|  | 4.6. **If N/PN to 4.3, 4.4 or 4.5**: Was an appropriate analysis used to estimate the effect of starting and adhering to the intervention? |  | NA |
|  | **Risk of bias judgement** |  | Low |

| **Bias due to missing data** | | | |
| --- | --- | --- | --- |
|  | 5.1 Were outcome data available for all, or nearly all, participants? |  | PY |
|  | 5.2 Were participants excluded due to missing data on intervention status? |  | N |
|  | 5.3 Were participants excluded due to missing data on other variables needed for the analysis? |  | PN |
|  | 5.4 **If PN/N to 5.1, or Y/PY to 5.2 or 5.3**: Are the proportion of participants and reasons for missing data similar across interventions? |  | NA |
|  | 5.5 **If PN/N to 5.1, or Y/PY to 5.2 or 5.3**: Is there evidence that results were robust to the presence of missing data? |  | NA |
|  | **Risk of bias judgement** |  | Low |

| **Bias in measurement of outcomes** | | | |
| --- | --- | --- | --- |
|  | 6.1 Could the outcome measure have been influenced by knowledge of the intervention received? |  | PN |
|  | 6.2 Were outcome assessors aware of the intervention received by study participants? |  | PY |
|  | 6.3 Were the methods of outcome assessment comparable across intervention groups? |  | Y |
|  | 6.4 Were any systematic errors in measurement of the outcome related to intervention received? |  | N |
|  | **Risk of bias judgement** |  | Moderate |

| **Bias in selection of the reported result** | | | |
| --- | --- | --- | --- |
|  | Is the reported effect estimate likely to be selected, on the basis of the results, from... |  |  |
|  | 7.1. ... multiple outcome *measurements* within the outcome domain? |  | PN |
|  | 7.2 ... multiple *analyses* of the intervention-outcome relationship? |  | PN |
|  | 7.3 ... different *subgroups*? |  | N |
|  | **Risk of bias judgement** |  | Low |

| **Overall bias** | | | |
| --- | --- | --- | --- |
|  | **Risk of bias judgement** |  | Moderate |

Effect of discharge opioid on persistent postoperative opioid use: a retrospective cohort study comparing tapentadol with oxycodone, Lam et al., 2022

|  | **Signalling questions** | **Description** | **Response options** |
| --- | --- | --- | --- |
| **Bias due to confounding** | | | |
|  | 1.1 Is there potential for confounding of the effect of intervention in this study?  **If N/PN to 1.1:** the study can be considered to be at low risk of bias due to confounding and no further signalling questions need be considered | Retrospective study design; mixed effects logistic regression analysis performed for other outcomes but not for rates of persistent postoperative opioid use between total modified-release and immediate-release opioid groups. | PY |
|  | **If Y/PY to 1.1**: determine whether there is a need to assess time-varying confounding: |  |  |
|  | 1.2. Was the analysis based on splitting participants’ follow up time according to intervention received?  **If N/PN**, answer questions relating to baseline confounding (1.4 to 1.6)  **If Y/PY**, go to question 1.3. |  | N |
|  | 1.3. Were intervention discontinuations or switches likely to be related to factors that are prognostic for the outcome?  **If N/PN**, answer questions relating to baseline confounding (1.4 to 1.6)  **If Y/PY**, answer questions relating to both baseline and time-varying confounding (1.7 and 1.8) |  | NA |

|  | **Questions relating to baseline confounding only** | | |
| --- | --- | --- | --- |
|  | 1.4. Did the authors use an appropriate analysis method that controlled for all the important confounding domains? | Logistic regression used to account for confounders, but was not performed to control for confounders relevant to outcome reported in the present review | PN |
|  | 1.5. **If Y/PY to 1.4**: Were confounding domains that were controlled for measured validly and reliably by the variables available in this study? |  | NI |
|  | 1.6. Did the authors control for any post-intervention variables that could have been affected by the intervention? |  | N |
|  | **Questions relating to baseline and time-varying confounding** | |  |
|  | 1.7. Did the authors use an appropriate analysis method that controlled for all the important confounding domains and for time-varying confounding? |  | NI |
|  | 1.8. **If Y/PY to 1.7**: Were confounding domains that were controlled for measured validly and reliably by the variables available in this study? |  | NA |
|  | **Risk of bias judgement** |  | Moderate |

| **Bias in selection of participants into the study** | | | |
| --- | --- | --- | --- |
|  | 2.1. Was selection of participants into the study (or into the analysis) based on participant characteristics observed after the start of intervention?  **If N/PN to 2.1:** go to 2.4 |  | N |
|  | 2.2. **If Y/PY to 2.1**: Were the post-intervention variables that influenced selection likely to be associated with intervention?  2.3 **If Y/PY to 2.2**: Were the post-intervention variables that influenced selection likely to be influenced by the outcome or a cause of the outcome? |  | NA  NA |
|  | 2.4. Do start of follow-up and start of intervention coincide for most participants? |  | Y |
|  | 2.5. **If Y/PY to 2.2 and 2.3, or N/PN to 2.4**: Were adjustment techniques used that are likely to correct for the presence of selection biases? |  | NA |
|  | **Risk of bias judgement** |  | Low |

| **Bias in classification of interventions** | | | |
| --- | --- | --- | --- |
|  | 3.1 Were intervention groups clearly defined? |  | Y |
|  | 3.2 Was the information used to define intervention groups recorded at the start of the intervention? |  | Y |
|  | 3.3 Could classification of intervention status have been affected by knowledge of the outcome or risk of the outcome? |  | PN |
|  | **Risk of bias judgement** |  | Low |

| **Bias due to deviations from intended interventions** | | | |
| --- | --- | --- | --- |
|  | **If your aim for this study is to assess the effect of assignment to intervention, answer questions 4.1 and 4.2** | |  |
|  | 4.1. Were there deviations from the intended intervention beyond what would be expected in usual practice? |  | N |
|  | 4.2. **If Y/PY to 4.1**: Were these deviations from intended intervention unbalanced between groups *and* likely to have affected the outcome? |  | NA |
|  | **If your aim for this study is to assess the effect of starting and adhering to intervention, answer questions 4.3 to 4.6** | |  |
|  | 4.3. Were important co-interventions balanced across intervention groups? |  | Y |
|  | 4.4. Was the intervention implemented successfully for most participants? |  | Y |
|  | 4.5. Did study participants adhere to the assigned intervention regimen? |  | Y |
|  | 4.6. **If N/PN to 4.3, 4.4 or 4.5**: Was an appropriate analysis used to estimate the effect of starting and adhering to the intervention? |  | NA |
|  | **Risk of bias judgement** |  | Low |

| **Bias due to missing data** | | | |
| --- | --- | --- | --- |
|  | 5.1 Were outcome data available for all, or nearly all, participants? |  | PY |
|  | 5.2 Were participants excluded due to missing data on intervention status? |  | N |
|  | 5.3 Were participants excluded due to missing data on other variables needed for the analysis? |  | PN |
|  | 5.4 **If PN/N to 5.1, or Y/PY to 5.2 or 5.3**: Are the proportion of participants and reasons for missing data similar across interventions? |  | NA |
|  | 5.5 **If PN/N to 5.1, or Y/PY to 5.2 or 5.3**: Is there evidence that results were robust to the presence of missing data? |  | NA |
|  | **Risk of bias judgement** |  | Low |

| **Bias in measurement of outcomes** | | | |
| --- | --- | --- | --- |
|  | 6.1 Could the outcome measure have been influenced by knowledge of the intervention received? |  | PN |
|  | 6.2 Were outcome assessors aware of the intervention received by study participants? |  | PY |
|  | 6.3 Were the methods of outcome assessment comparable across intervention groups? |  | Y |
|  | 6.4 Were any systematic errors in measurement of the outcome related to intervention received? |  | N |
|  | **Risk of bias judgement** |  | Moderate |

| **Bias in selection of the reported result** | | | |
| --- | --- | --- | --- |
|  | Is the reported effect estimate likely to be selected, on the basis of the results, from... |  |  |
|  | 7.1. ... multiple outcome *measurements* within the outcome domain? |  | PN |
|  | 7.2 ... multiple *analyses* of the intervention-outcome relationship? |  | PN |
|  | 7.3 ... different *subgroups*? |  | N |
|  | **Risk of bias judgement** |  | Low |

| **Overall bias** | | | |
| --- | --- | --- | --- |
|  | **Risk of bias judgement** |  | Moderate |

Effect of prolonged-released oxycodone/naloxone in postoperative pain management after total knee replacement: a nonrandomized prospective trial, Oppermann et al., 2016

|  | **Signalling questions** | **Description** | **Response options** |
| --- | --- | --- | --- |
| **Bias due to confounding** | | | |
|  | 1.1 Is there potential for confounding of the effect of intervention in this study?  **If N/PN to 1.1:** the study can be considered to be at low risk of bias due to confounding and no further signalling questions need be considered |  | Y |
|  | **If Y/PY to 1.1**: determine whether there is a need to assess time-varying confounding: |  |  |
|  | 1.2. Was the analysis based on splitting participants’ follow up time according to intervention received?  **If N/PN**, answer questions relating to baseline confounding (1.4 to 1.6)  **If Y/PY**, go to question 1.3. |  | N |
|  | 1.3. Were intervention discontinuations or switches likely to be related to factors that are prognostic for the outcome?  **If N/PN**, answer questions relating to baseline confounding (1.4 to 1.6)  **If Y/PY**, answer questions relating to both baseline and time-varying confounding (1.7 and 1.8) |  | NA |

|  | **Questions relating to baseline confounding only** | | |
| --- | --- | --- | --- |
|  | 1.4. Did the authors use an appropriate analysis method that controlled for all the important confounding domains? |  | PN |
|  | 1.5. **If Y/PY to 1.4**: Were confounding domains that were controlled for measured validly and reliably by the variables available in this study? |  | NI |
|  | 1.6. Did the authors control for any post-intervention variables that could have been affected by the intervention? |  | N |
|  | **Questions relating to baseline and time-varying confounding** | |  |
|  | 1.7. Did the authors use an appropriate analysis method that controlled for all the important confounding domains and for time-varying confounding? |  | NI |
|  | 1.8. **If Y/PY to 1.7**: Were confounding domains that were controlled for measured validly and reliably by the variables available in this study? |  | NA |
|  | **Risk of bias judgement** |  | Moderate |

| **Bias in selection of participants into the study** | | | |
| --- | --- | --- | --- |
|  | 2.1. Was selection of participants into the study (or into the analysis) based on participant characteristics observed after the start of intervention?  **If N/PN to 2.1:** go to 2.4 | Selection not altered after start of intervention | N |
|  | 2.2. **If Y/PY to 2.1**: Were the post-intervention variables that influenced selection likely to be associated with intervention?  2.3 **If Y/PY to 2.2**: Were the post-intervention variables that influenced selection likely to be influenced by the outcome or a cause of the outcome? |  | NA  NA |
|  | 2.4. Do start of follow-up and start of intervention coincide for most participants? |  | Y |
|  | 2.5. **If Y/PY to 2.2 and 2.3, or N/PN to 2.4**: Were adjustment techniques used that are likely to correct for the presence of selection biases? |  | NA |
|  | **Risk of bias judgement** |  | Low |

| **Bias in classification of interventions** | | | |
| --- | --- | --- | --- |
|  | 3.1 Were intervention groups clearly defined? |  | Y |
|  | 3.2 Was the information used to define intervention groups recorded at the start of the intervention? |  | Y |
|  | 3.3 Could classification of intervention status have been affected by knowledge of the outcome or risk of the outcome? |  | PN |
|  | **Risk of bias judgement** |  | Low |

| **Bias due to deviations from intended interventions** | | | |
| --- | --- | --- | --- |
|  | **If your aim for this study is to assess the effect of assignment to intervention, answer questions 4.1 and 4.2** | |  |
|  | 4.1. Were there deviations from the intended intervention beyond what would be expected in usual practice? |  | N |
|  | 4.2. **If Y/PY to 4.1**: Were these deviations from intended intervention unbalanced between groups *and* likely to have affected the outcome? |  | NA |
|  | **If your aim for this study is to assess the effect of starting and adhering to intervention, answer questions 4.3 to 4.6** | |  |
|  | 4.3. Were important co-interventions balanced across intervention groups? |  | NA |
|  | 4.4. Was the intervention implemented successfully for most participants? |  | NA |
|  | 4.5. Did study participants adhere to the assigned intervention regimen? |  | NA |
|  | 4.6. **If N/PN to 4.3, 4.4 or 4.5**: Was an appropriate analysis used to estimate the effect of starting and adhering to the intervention? |  | NA |
|  | **Risk of bias judgement** |  | Low |

| **Bias due to missing data** | | | |
| --- | --- | --- | --- |
|  | 5.1 Were outcome data available for all, or nearly all, participants? |  | PY |
|  | 5.2 Were participants excluded due to missing data on intervention status? |  | N |
|  | 5.3 Were participants excluded due to missing data on other variables needed for the analysis? |  | PN |
|  | 5.4 **If PN/N to 5.1, or Y/PY to 5.2 or 5.3**: Are the proportion of participants and reasons for missing data similar across interventions? |  | NA |
|  | 5.5 **If PN/N to 5.1, or Y/PY to 5.2 or 5.3**: Is there evidence that results were robust to the presence of missing data? |  | NA |
|  | **Risk of bias judgement** |  | Low |

| **Bias in measurement of outcomes** | | | |
| --- | --- | --- | --- |
|  | 6.1 Could the outcome measure have been influenced by knowledge of the intervention received? |  | PN |
|  | 6.2 Were outcome assessors aware of the intervention received by study participants? |  | Y |
|  | 6.3 Were the methods of outcome assessment comparable across intervention groups? |  | Y |
|  | 6.4 Were any systematic errors in measurement of the outcome related to intervention received? |  | N |
|  | **Risk of bias judgement** |  | Moderate |

| **Bias in selection of the reported result** | | | |
| --- | --- | --- | --- |
|  | Is the reported effect estimate likely to be selected, on the basis of the results, from... |  |  |
|  | 7.1. ... multiple outcome *measurements* within the outcome domain? |  | PN |
|  | 7.2 ... multiple *analyses* of the intervention-outcome relationship? |  | PN |
|  | 7.3 ... different *subgroups*? |  | N |
|  | **Risk of bias judgement** |  | Low |

| **Overall bias** | | | |
| --- | --- | --- | --- |
|  | **Risk of bias judgement** |  | Moderate |

1. 80% drop out is given as an example here a different proportion can be used depending on the context of the systematic review area [↑](#footnote-ref-1)
2. 80% drop out is given as an example here a different proportion can be used depending on the context of the systematic review area [↑](#footnote-ref-2)
3. 80% drop out is given as an example here a different proportion can be used depending on the context of the systematic review area [↑](#footnote-ref-3)
4. Reviewers may choose to use estimates from a subgroup analysis which may explain the inconsistency but should be cautious that such a explanation of heterogeneity may be due to the play of chance [↑](#footnote-ref-4)
5. This reflects GRADE guidance that guideline developers may use a less stringent threshold for judging imprecision of an intervention’s benefits when there is no evidence of harm compared with when judging the benefits of an intervention where there is strong evidence of harm [↑](#footnote-ref-5)
6. 80% drop out is given as an example here a different proportion can be used depending on the context of the systematic review area [↑](#footnote-ref-6)
7. 80% drop out is given as an example here a different proportion can be used depending on the context of the systematic review area [↑](#footnote-ref-7)
8. 80% drop out is given as an example here a different proportion can be used depending on the context of the systematic review area [↑](#footnote-ref-8)
9. Reviewers may choose to use estimates from a subgroup analysis which may explain the inconsistency but should be cautious that such a explanation of heterogeneity may be due to the play of chance [↑](#footnote-ref-9)
10. This reflects GRADE guidance that guideline developers may use a less stringent threshold for judging imprecision of an intervention’s benefits when there is no evidence of harm compared with when judging the benefits of an intervention where there is strong evidence of harm [↑](#footnote-ref-10)
